# *Polygonum cuspidatum* inhibits the growth of osteosarcoma cells via impeding Akt/ERK/EGFR signaling pathways

**DOI:** 10.1080/21655979.2021.2017679

**Published:** 2022-02-07

**Authors:** Jun Zhao, Boyu Pan, Xinglu Zhou, Chunnuan Wu, Fengcheng Hao, Jie Zhang, Liren Liu

**Affiliations:** a Department of Bone & Soft Tissue Tumor, Tianjin Medical University Cancer Institute & Hospital, National Clinical Research Center for Cancer, Key Laboratory of Cancer Prevention and Therapy, Tianjin’s Clinical Research Center for Cancer, Tianjin, China; b Department of Gastrointestinal Cancer Biology, Tianjin Medical University Cancer Institute & Hospital, National Clinical Research Center for Cancer, Key Laboratory of Cancer Prevention and Therapy, Tianjin’s Clinical Research Center for Cancer, Tianjin, China; c Department of Pharmacy, Tianjin Medical University Cancer Institute & Hospital, National Clinical Research Center for Cancer, Key Laboratory of Cancer Prevention and Therapy, Tianjin’s Clinical Research Center for Cancer, Tianjin, China; d Department of General Surgery, People’s Hospital of Zoucheng City, Jining, Shandong, China

**Keywords:** *Polygonum cuspidatum*, bioinformatics, Osteosarcoma (OS), network pharmacology, AKt/ERK/EGFR signaling pathways, Chinese Herb Medicine (CHM)

## Abstract

Osteosarcoma (OS), the most prevalent bone malignancy, mainly affects children and adolescents. Despite recent advances in multimodal therapy, the overall survival rate for OS patients remains poor. Chinese herb medicine (CHM) is an alternative therapeutic option for multifaceted diseases such as malignant tumors. For centuries in China, *polygonum cuspidatum*, a classic CHM, has been used to treat several diseases, nevertheless, the mechanisms underlying its therapeutic effects have not been fully elucidated. Through network pharmacology and bioinformatic tools, we evaluated the pharmacological activity of *polygonum cuspidatum*. We found that it has a potential therapeutic effect on malignant tumors, which was subsequently verified by intragastric administration of *polygonum cuspidatum* to OS cell xenografted mice models. Next, to establish the action mechanism of *polygonum cuspidatum*, we built a disease/drug-target PPI network that was made up of 250 core treatment targets against OS using Cytoscape software. Enrichment evaluation for the above targets indicated that *polygonum cuspidatum* may exert its effects on the cell cycle and apoptosis of OS cells through inhibiting Akt/ERK/EGFR pathways. Finally, the above *in silico* results were experimentally validated via a series of molecular biological and cell functional analyses. Taken together, the findings show that *polygonum cuspidatum* has a significant potential for OS treatment, which provides a novel insight into the discovery of CHM-based drug against malignancies.

**Abbreviations:**

CC: Closeness centrality; OS: Osteosarcoma; TCM: Traditional Chinese medicine; NSCLC: Non-small cell lung cancer; DC: Degree centrality; CHM: Chinese herb medicine; BC: Betweenness centrality

## 1. Introduction

Globally, osteosarcoma (OS), the most prevalent primary malignant tumor of the bone, particularly affects children and adolescents [1–3]. Over the past decades, although therapeutic strategy for OS has been improved, such as surgical resection combined with adjuvant chemotherapy or radiotherapy, the clinical prognosis of OS is poor while the 5-year survival rate of OS patients with distal metastasis and is approximately 20% [4–8]. Therefore, it is imperative to develop novel therapeutic drugs and strategies to improve the outcome of this deadly disease among adolescents.

CHM, as an important part of traditional Chinese medicine (TCM), has been widely used in East and Southeast Asia since ancient civilization [9]. Recent studies have shown that CHM could indeed offer a promising perspective for treating malignant diseases with its unique clinical effects [10,11]. As a complex system, CHM is generally composed of multiple ingredients, and each ingredient has multiple targets. Hence, the complex chemical composition as well as the complexity of the interactions among them makes it a huge challenge to reveal the scientific basis of CHMs, which greatly hinders the acceptance of CHM worldwide [12]. In recent years, due to the advances in bioinformatics, systems biology, and polypharmacology, network-based pharmacology was launched and employed as a powerful tool for cost-effective drug discovery, facilitating the paradigm shift from ‘one drug, one target, one disease’ to ‘drug-target-disease network’ [13–16]. In line with the holistic theory of TCM, TCM network pharmacology has made a great contribution to the CHM study, such as screening of active ingredients, prediction of potential drug targets, which paves a new path for revealing the action mechanism of CHM, and significantly accelerates the process of new drug discovery [17,18].

Currently, *polygonum cuspidatum* is used as folk medicines for treating several diseases, including atherosclerosis, hypertension and diabetes [19,20]. Furthermore, the extracts of *polygonum cuspidatum* has also shown therapeutic effects on malignant diseases, such as lung and breast cancers [21,22]. However, the scientific basis of *polygonum cuspidatum* against osteosarcoma has yet to be systemically elucidated. The current study aims to explore the potential core targets and pathways of *polygonum cuspidatum* against osteosarcoma by using a TCM network pharmacology approach. The subsequent *in vitro* assays guided by *in silico* analyses revealed a direct suppressive effect of *polygonum cuspidatum* on OS cell growth through simultaneously disturbance of multiple signaling pathways. Hence, exemplified by a mechanism-of-action study on *polygonum cuspidatum*, our results show that the TCM network pharmacology coupled with bioinformatics-guided study is a promising approach to explore the scientific basis of CHM against refractory diseases.

## 2. Materials and methods

### 2.1. Cell culture and reagents

Human OS U2OS as well as HOS cell lines were purchased from the China Infrastructure of Cell Line Resources. Respectively, they were cultured in 10% (v/v) FBS (fetal bovine serum, Thermofisher)-supplemented DMEM and MEM media containing 100 U/ml penicillin/streptomycin in the humidified 5% CO_2_ atmosphere at 37°C. The apoptosis detection and BrdU Flow kits were procured from BD Biosciences Pharmingen (USA). Antibodies against Bax, Bcl-2, beta-actin Cyclin D1, Caspase 9, were acquired from Proteintech, and the anti-Cyclin B1, p-Akt (T308/S473), Cyclin A2, pan-ERK, p-ERK (T202/Y204), pan-Akt/EGFR antibodies were purchased from CST. The drug standard substance of *polygonum cuspidatum* (ID: MG7G-H081) was obtained from China National Institutes for Food and Drug Control. *Polygonum cuspidatum* was used with 0.05 g/ml as the final concentration. Meanwhile, the above concentration was filtered through 0.22 µm pores, sterilized, and thereafter diluted for further biological studies.

### 2.2. *Candidate ingredients composition of* polygonum cuspidatum

According to our previous work, seven key ingredients with high content in *polygonum cuspidatum* were identified by UPLC-MS/MS, namely quercetin (MOL000098), polydatin (MOL013289), resveratrol (MOL012744), emodin (MOL000472), apigenin (MOL000008), rhein (MOL002268) and catechin (MOL000492) [23]. The MOL number of these ingredients were bought from the TCM Systems Pharmacology (TCMSP) Database.

### 2.3. The drug/disease target identification, protein–protein interaction network construction and enrichment analysis

To establish the probable drug targets for medicinal compositions of *polygonum cuspidatum*, the systematic drug targeting approach was employed. The candidate drug targets of above 7 ingredients were obtained from TCMSP database (data not shown), and these drug targets will be considered as targets for *polygonum cuspidatum*. Meanwhile, the OS-related targets were acquired from GEO database. Three gene expression datasets (GSE11414, GSE12865 and GSE16088) were included. Bisogenet, an important plugin of Cytoscape software, was utilized to analyze the protein-protein interaction (PPI) data. Interaction networks for candidate drug targets of 7 ingredients from *polygonum cuspidatum* and the obtained OS-related targets were constructed using Bisogenet plugin and visualized using Cytoscape. The detailed procedure of topology parameter calculation for the nodes in network and the functional as well as pathway enrichment assessments of core targets had been described previously [24].

### 2.4. Cell function and molecular biological analyses

Cell viabilities and growth abilities were evaluated by the CCK-8 kit following manufacturer's protocol. Wound-healing assay was performed, and after washing using phosphate-buffered saline, the drug solution was added to the wells and then were incubated for 24 h. Cell colony formation and morphology assays were conducted as previously described [24]. Cell cycle assay and apoptosis evaluation were based on the manufacturer’s protocol.

### 2.5. Mouse work

BALB/c nude mice (4-week-old male) were bought from Beijing Vital River Laboratory Animal Technology Co., Ltd. (China). The Animal Ethics Committee of Tianjin Medical University Cancer Institute & Hospital approved the use of animals in this study. When mice were 6 weeks of age, U2OS cells (1.5 × 10^6^/100 μL) were administered subcutaneously into the left flank of 14 mice. After 10 days, tumors could be seen by the naked eye, when mice were daily gavaged (p.o.) with either *polygonum cuspidatum* (300 mg/kg weight) or the same volume of normal saline (control). These 14 mice were randized into 2 groups, 7 mice for each group. Meanwhile, daily evaluations of tumor volumes were done via the formula as: long diameter × (short diameter)^2^/2. Mice were sacrificed on day 14 after which the tumor tissues were resected and weighed. No death occurred during the experiment. The immunohistochemical experiments were conducted as previously described [24].

### 2.6. Molecular docking

CB-Dock online software was employed to predict the binding activities between proteins and compounds as well as to calculate the center and cavity size [25]. Ligand files in SDF formats and core protein targets in PDB formats were inputted to CB-Dock to, respectively, assess binding activities.

### 2.7. Statistical analysis

Data were analyzed via SPSS 17.0 (USA) and were expressed as means ± S.D. Between-group differences were estimated by the Student’s *t*-test, and *p* < 0.05 was the cutoff for significance.

## 3. Results

The therapeutic effects and mechanism-of-action of *polygonum cuspidatum* against osteosarcoma have not been comprehensively elucidated. Here, we used a TCM network pharmacology approach to explore the potential core targets and signaling pathways involved in osteosarcoma cells upon *polygonum cuspidatum* treatment. Furthermore, experimental assays were carried out to validate the effect and mechanism-of-action of *polygonum cuspidatum* on osteosarcoma cells both *in vivo* and *in vitro*.

### 3.1. *Drug target screening and enrichment analyses for the key ingredients in* polygonum cuspidatum

**Figure 1.**
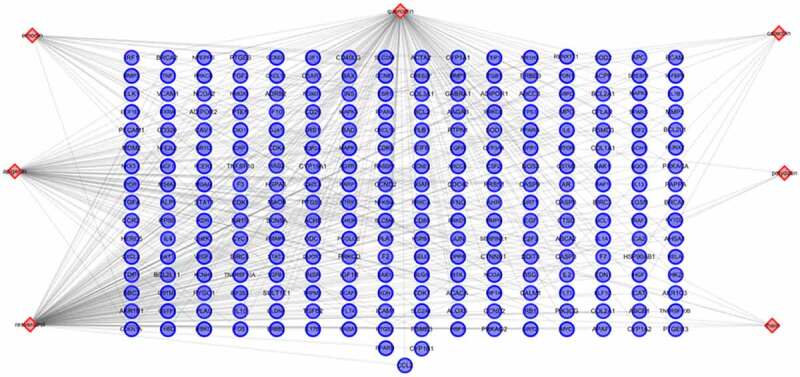
*Construction of the* polygonum cuspidatum important *ingredients and drug targets network*. The network was established by connecting the candidate key ingredients in polygonum cuspidatum and their putative targets.

We have previously identified seven key ingredients with high content in *polygonum cuspidatum* by UPLC-MS/MS, such as quercetin, resveratrol, polydatin, emodin, apigenin, catechin and rhein [23]. In this study, we first explored the probable drug targets of *polygonum cuspidatum* using TCMSP database, and obtained 260 putative targets for these compounds (Figure 1). Intriguingly, many drug targets were overlapped among these ingredients, which suggests that these ingredients may have similar or synergistic biological effects on OS cells.

**Figure 2.**
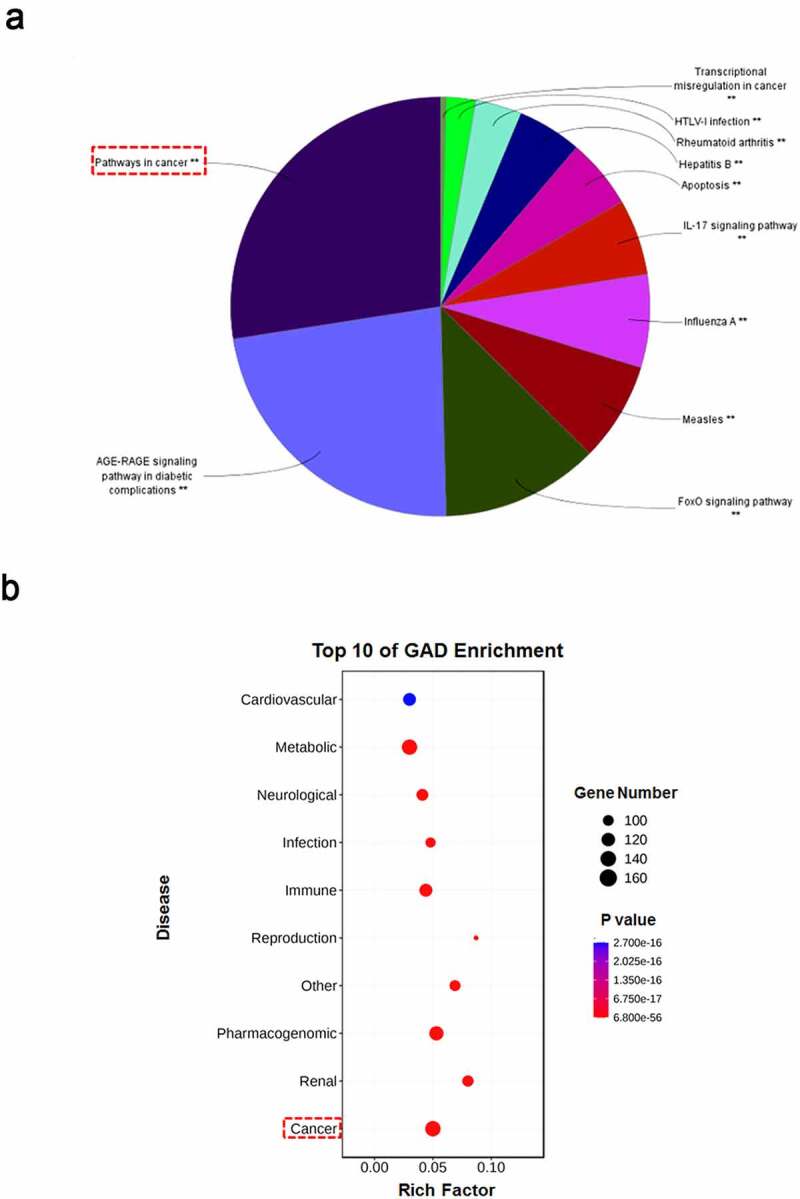
*Enrichment analysis for putative targets of* polygonum cuspidatum. (a) Drug targets enriched in signaling pathways were performed using DAVID v6.8 (*p* < 0.05). (b) Drug targets enriched in representative diseases were performed using DAVID v6.8 (*p* < 0.05).

Having obtained the drug targets of *polygonum cuspidatum*, we next used DAVID v6.8 to conduct KEGG signaling enrichment analysis for the 260 targets. Interestingly, the most enriched signaling pathways by *polygonum cuspidatum* was Pathways in cancer, suggesting probable effects of *polygonum cuspidatum* on tumor treatment (Figure 2(a)). To further verify the potential significance of *polygonum cuspidatum* in tumor therapy, we conducted disease enrichment evaluation for *polygonum cuspidatum* by using GAD Disease Class (DAVID v6.8). ‘Cancer’ was first among the top-enriched diseases (Figure 2(b)). Together, these results imply therapeutic potential of *polygonum cuspidatum* for cancer.

### 3.2. Identification of osteosarcoma-related targets

**Figure 3.**
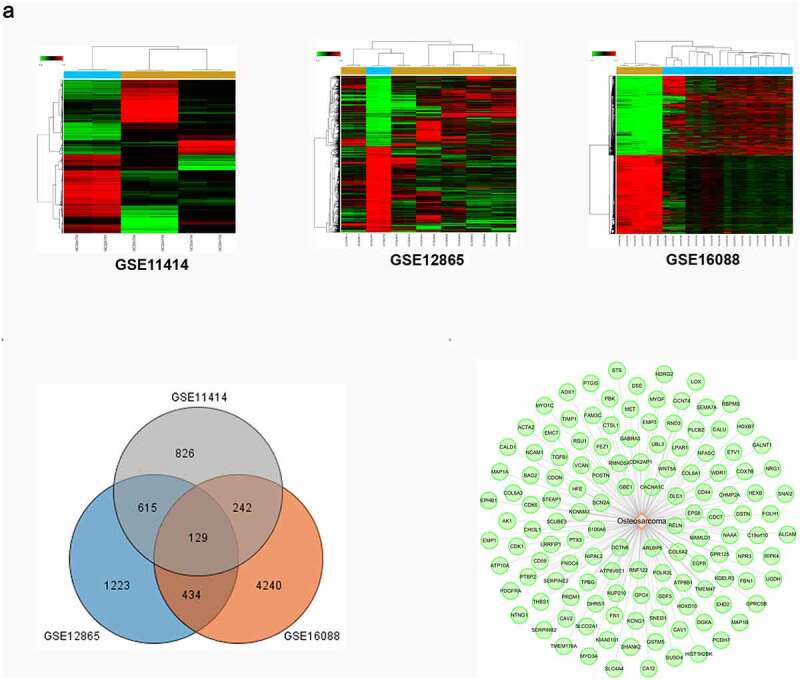
*OS-related disease targets were collected from the GEO database*. (a) Heat maps from GEO chips, including GSE11414, GSE12865 and GSE16088. (b) Venn diagram composed of 129 overlapped OS-related targets from 3 GEO chips. (c) OS-associated disease targets network construction.

According to the TCM theory, OS is mostly manifested as the syndromes of blood stasis. Given the effect of *polygonum cuspidatum* on promoting blood circulation to remove blood stasis, we assessed the potential treatment effects of *polygonum cuspidatum* on OS. Three datasets of gene expressions (GSE11414, GSE12865 and GSE16088) of OS tissues as well as the corresponding normal tissues were collected from Gene Expression Omnibus (GEO) database. Then, 129 disease targets which were overlapping among the datasets were obtained as ‘OS-related targets’ for subsequent analyses (Figure 3(a–c)).

### 3.3. *Protein–protein interaction networks construction and enrichment analyses for core therapeutic targets of* polygonum cuspidatum *in osteosarcoma*

**Figure 4.**
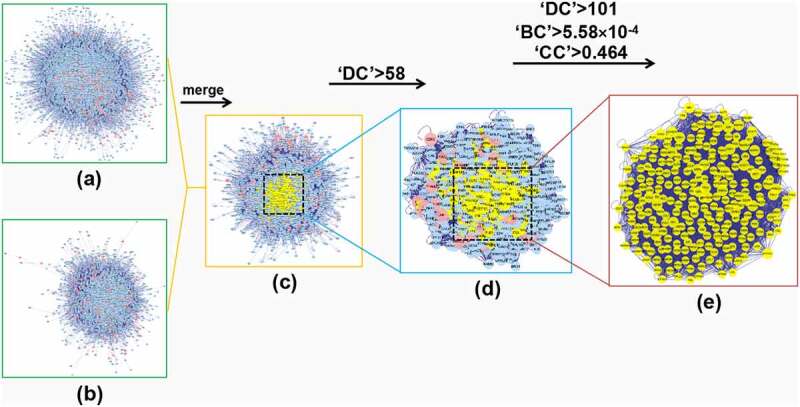
*In silico evaluation and network construction of the core targets for* polygonum cuspidatum *in OS*. (a) Drug target PPI network of polygonum cuspidatum was composed of 7514 nodes and 162758 edges. (b) PPI network of OS-associated disease targets was made of 3785 nodes and 86465 edges. (c) Integrative PPI network of polygonum cuspidatum in OS was made up of 2939 nodes and 73588 edges. (d) PPI network of the key targets extracted from c, in which 781 nodes and 33351 edges was included. (e) PPI network containing the core targets extracted from d, including 250 nodes and 7994 edges were shown.

To have a better understanding of the complicated association among these targets, we then developed a PPI network composed of putative drug-targets for *polygonum cuspidatum*, containing 7514 nodes and 162758 edges. Meanwhile, a PPI network of OS-related target, which contains 3785 nodes as well as 86465 edges, was also build via the same method. To reveal the pharmacological mechanism of *polygonum cuspidatum* in OS, we then intersected the above 2 networks, and thus obtained 2939 nodes and 73588 edges. According previous reports, topology parameters for every node in the above network were determined, and a network comprising significant targets for *polygonum cuspidatum* in OS was thus constructed, which contains 781 nodes and 33351 edges [26]. To establish key targets, we used 3 topological parameters (DC, CC and BC) to cut down the number of node (core target) with stringent values, leading to 250 core nodes/targets (Figure 4).

**Figure 5.**
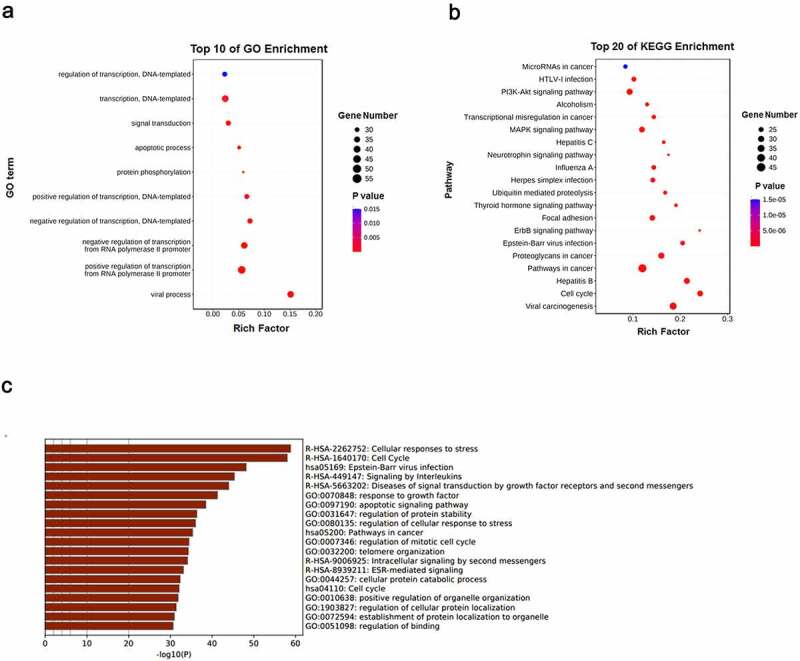
*GO and KEGG enrichment analyses of the core targets for* polygonum cuspidatum *against OS*. (a) & (b) The core targets that were enriched in different biological processes (GO-BP) and signaling pathways (KEGG) as assessed by DAVID v6.8 (*p*-value < 0.05). (c) The core targets that were enriched different biological processes and signaling pathways as assessed by Metascape (*p*-value < 0.05).

GO biological process and KEGG signaling pathways analyses for the identified 250 core targets were subsequently performed. Our results showed that the related enriched biological processes were in transcription and apoptosis, while the pathways were involved in cancer, MAPK signaling pathway, viral carcinogenesis, PI3K-Akt signaling pathway, ErbB signaling pathway, and so on (Figure 5(a–c)). These findings indicated that *polygonum cuspidatum* inhibits cell growth by deregulating critical pathways related to cell cycle, proliferation and apoptosis in OS cells.

### 3.4. Orally administered polygonum cuspidatum suppressed the growth of xenografted osteosarcoma cells on mice

**Figure 6.**
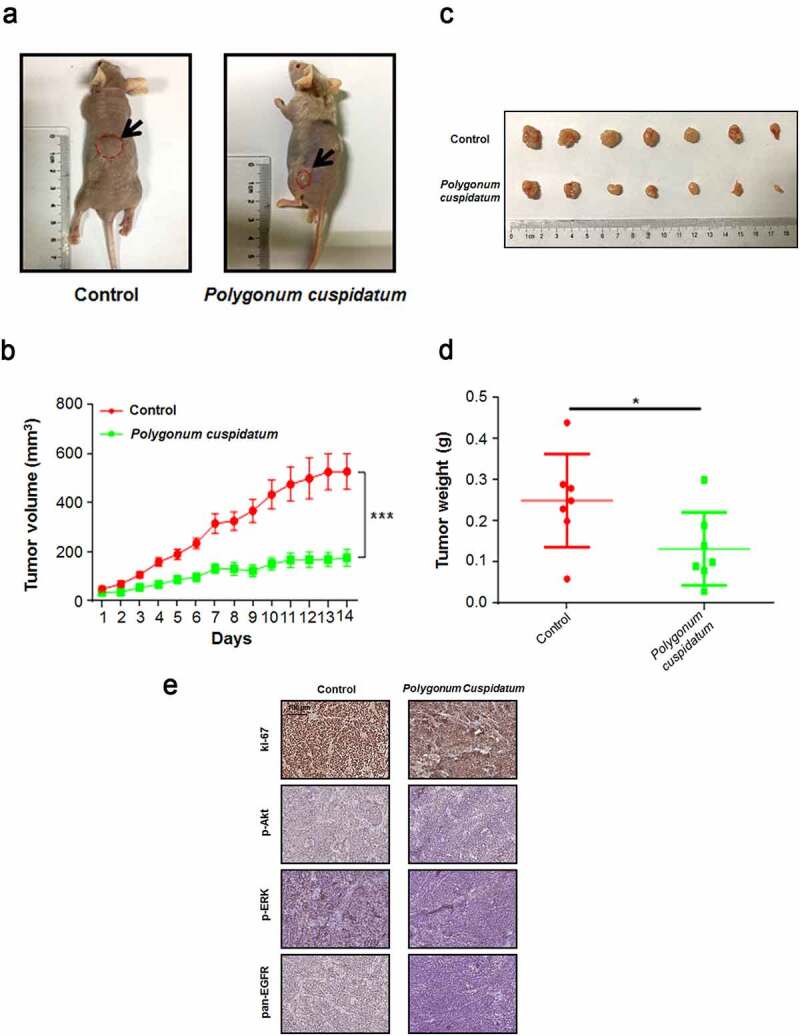
*Polygonum cuspidatum inhibited the proliferation of xenografted osteosarcoma cells on Balb/c nude mice*. (a) The photos of representative tumor masses of drug-treatment and control groups were presented. (b) Tumor volumes were determined once daily for 14 successive days. ***p < 0.001 by the Student’s *t*-test. (c) The photos of tumor sizes were shown after tumor removal at end of the experiment. (d) The average tumor weights of two groups were determined after the tumors were resected and weighted on the day 14. *p < 0.05 by Student’s *t*-test. (e) Immunohistochemistry staining for ki-67, p-Akt, p-ERK and pan-EGFR were performed by using the tumor slides from control and polygonum cuspidatum-treated groups.

To verify the putative anti-tumor effect of *polygonum cuspidatum*, we orally administrated the drug to xenografted OS cell-bearing mice. Figure 6(a) shows that growth of the tumor on mice was significantly suppressed by *polygonum cuspidatum* treatment. At day 14th, the average tumor volume of *polygonum cuspidatum* treated group was approximately 3-fold (*p* < 0.001) smaller than that of control group (Figure 6(b)). Consistently, the tumor weights between two groups also showed a dramatic difference (*p* < 0.05) (Figure 6(c,d)). The immunohistochemical analysis showed that the number of Ki-67, p-Akt, p-ERK and pan-EGFR positive cells was significantly reduced in the *polygonum cuspidatum* treatment group, respectively (Figure 6(e)). Above these results convincingly showed that *polygonum cuspidatum* has a direct anti-tumor effect on OS cells *in vivo*.

### 3.5. *Polygonum cuspidatum* suppressed cell growth, motility, and viability of osteosarcoma cells

**Figure 7.**
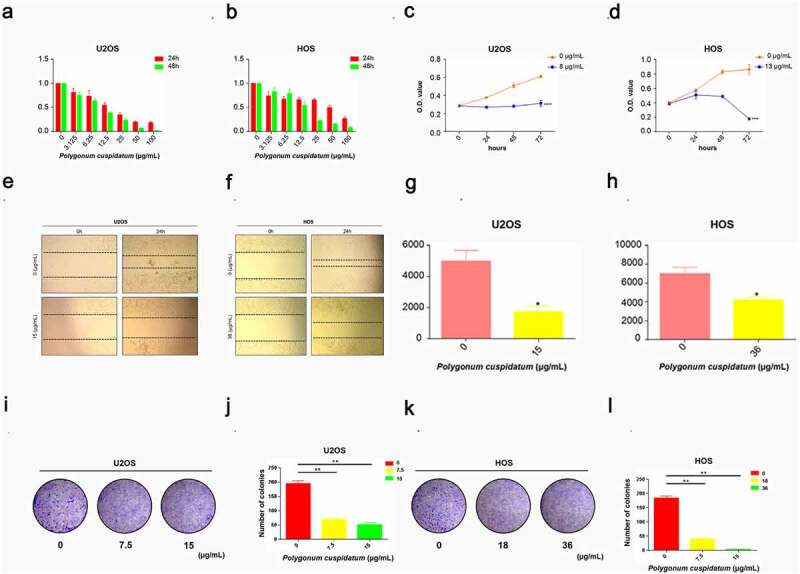
Polygonum cuspidatum *inhibited OS cells growth and suppressed its viability*. (a) & (b) The CCK-8 assay was carried out to evaluate OS cell viability upon polygonum cuspidatum treatments with different dosages (0,3.125,6.25,12.5,25,50,100 μg/mL) in 24 h and 48 h. (c) & (d) CCK-8 assay was conducted to assess OS cell growth ability after treating with polygonum cuspidatum at different dosages (8 or 13 μg/mL) in 24 h, 48 h and 72 h. (e–h) Wound-healing assay was used to evaluate the cell motility after polygonum cuspidatum treatment for 24 h. *p < 0.05 by Student’s *t*-test. (i–l) Cell colony formation assay was carried out to access the cell clonality after polygonum cuspidatum treatment for 24 h. **p < 0.01 was based on the Student’s *t*-test.

To verify the above *in silico* analysis result, we then evaluated the pharmacological effect of *polygonum cuspidatum* on OS cell lines. The result of CCK-8 analysis revealed that the growth and viability of U2OS as well as HOS cells were both dose- and time-dependently markedly suppressed by *polygonum cuspidatum* (Figure 7(a–d)). The IC_50_ analysis result indicated that *polygonum cuspidatum* exerted 50% suppressive effects on U2OS as well as HOS cells at 15.13 ± 0.41 μg/mL, 8.73 ± 0.15 μg/mL and 36.66 ± 3.86 μg/mL, 13.42 ± 2.12 μg/mL after treated for 24 and 48 hours, respectively. Based on these IC_50_ values, we then evaluate the drug effects of *polygonum cuspidatum* on these OS cells using wound-healing assay. It was shown that *polygonum cuspidatum* markedly suppressed OS cell migration, relative to control group (Figure 7(e–h)). Furthermore, colony forming ability of the OS cells was determined, and our result showed that OS cells exhibited a dose-dependent decrease in cell clonality with the addition of *polygonum cuspidatum* (Figure 7(i–l)). Thus, *polygonum cuspidatum* has a direct downregulation effect on OS cell growth, motility as well as viability *in vitro*.

### 3.6. *Polygonum cuspidatum* initiated apoptosis and S-phase cell cycle arrest in osteosarcoma cells

**Figure 8.**
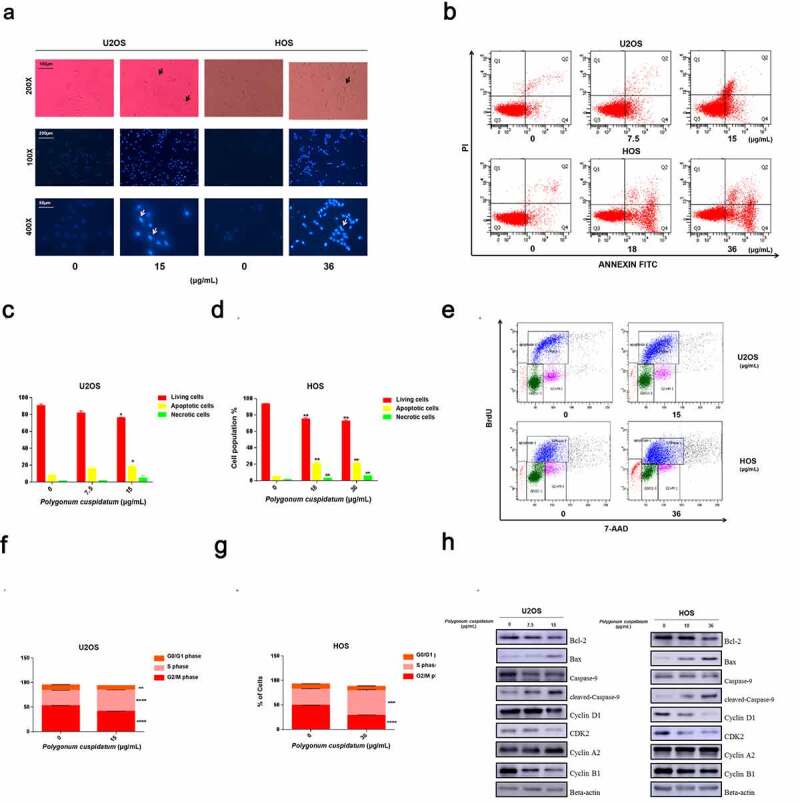
Polygonum cuspidatum *treatment led to apoptosis and disturbance of cell cycle in OS cells*. (a) Cell morphology was observed in white light and fluorescence field. (b) Induced apoptosis of U2OS and HOSOS cells upon polygonum cuspidatum treatment. Apoptotic events were evaluated by flow cytometry after U2OS and HOS cells were treated with polygonum cuspidatum at varying doses (U2OS: 0, 7.5 and 15 μg/mL, HOS: 0, 18 and 36 μg/mL) for 24 h. (c) & (d) Statistical analyses of apoptotic rates in U2OS and HOS cells. **p < 0.01, by Student’s *t*-test. (e) Cell cycle assessment after polygonum cuspidatum treatment in U2OS (0 and 15 μg/mL) and HOS (0 and 36 μg/mL) for 24 h by using flow cytometry. (f) & (g) Statistical analysis of the proportions of the cells in different phases using U2OS and HOS cells. **p < 0.01, based on the Student’s *t*-test. (h) OS cells were treated with polygonum cuspidatum at different concentrations(U2OS: 0, 7.5 and 15 μg/mL; HOS: 0, 18 and 36 μg/mL) for 24 h. Expression levels of cleaved-caspase-9, Bcl-2, pan-caspase-9, Bax, CyclinA2, Cyclin D1, CDK2, and CyclinB1 were analyzed by Western blot.

To elucidate on the action mode of *polygonum cuspidatum* on OS cells, we next conducted a serial of cellular functional assays. Firstly, we observed the cell morphology after treating OS cells with *polygonum cuspidatum* for 24 h. Relative to control cells, *polygonum cuspidatum*-treated cells exhibited typical characteristics of apoptosis, including disappearance of stereopsis and cell shrinkage. Consistently, dense Hoechst 33,342 staining of the cell nucleus was confirmed by fluorescence microscopy (Figure 8(a)). Furthermore, flow cytometry assessment result showed that cell population underwent apoptosis was significantly dose-dependently increased after *polygonum cuspidatum* treatment (Figure 8(b–d)). The following Western blotting results also demonstrated that *polygonum cuspidatum* could dose-dependently up-regulate the levels of cleaved Caspase-9 and Bax pro-apoptosis proteins, while down-regulated anti-apoptosis protein Bcl-2 level (Figure 8(h) and Supplementary Figure S2). Moreover, BrdU-incorporing OS cell profiling was conducted to assess the effects of *polygonum cuspidatum* on cell cycle. Our result indicated that *polygonum cuspidatum* could significantly inhibit the proliferation rate by arresting OS cells at S phase (Figure 8(e–g)). In line with this, such as cyclin B1, Cyclin D1 and CDK 2 levels were down-regulated, whereas Cyclin A2, the S phase-specific marker, was accumulated in *polygonum cuspidatum*-treated OS cells (Figure 8(h) and Supplementary Figure S2). These findings suggested that *polygonum cuspidatum* could suppress OS cell growth by promoting apoptosis and arresting OS cell cycle at S phase.

### 3.7. *Growth inhibition of osteosarcoma cells* by polygonum cuspidatum *through impeding Akt/ERK/EGFR pathways*

**Figure 9.**
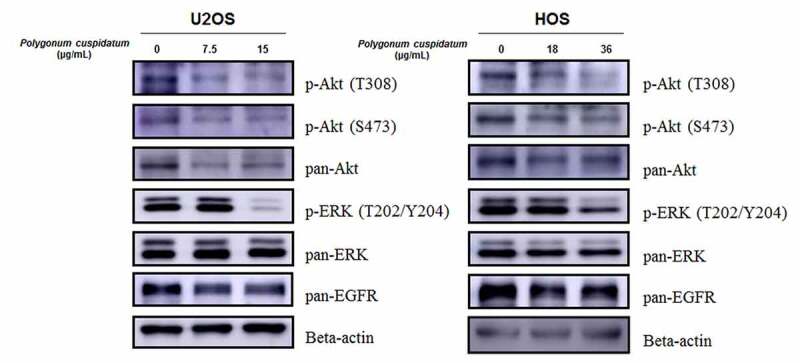
D*own-regulation of p-Akt, p-ERK and EGFR levels by p*olygonum cuspidatum *treatment*. OS cells were treated with polygonum cuspidatum at varying doses (U2OS: 0, 7.5 and 15 μg/mL; HOS: 0, 18 and 36 μg/mL) for 24 h. The levels of p-Akt (T308 &S473), pan-Akt, p-ERK (T202/Y204), pan-ERK and pan-EGFR were evaluated by Western blotting.

To investigate the action mechanisms of suppressive effects of *polygonum cuspidatum* on OS cell proliferation, we then determined the status of some key pathways involved in cell viability and proliferation. According to previous KEGG enrichment analysis result, MAPK, PI3K-Akt as well as ErbB pathways were chosen for subsequent investigations. As expected, our Western blotting result showed that these pathways were significantly impeded by *polygonum cuspidatum*, as levels of key factors in these pathways, including p-ERK (T202/Y204) and p-Akt (T308 & S473), were dramatically decreased. Meanwhile, the level of pan-EGFR was also down-regulated upon *polygonum cuspidatum* treatment (Figure 9 and Supplementary Figure S3). Together, these results indicated that the impaired apoptotic and proliferative processes in OS cells is attributed to simultaneous inactivation of Akt/ERK/EGFR pathways, underscoring the ‘multi-ingredient, multi-target as well as multi-function’ characteristics of *polygonum cuspidatum* as a CHM.

### 3.8. Evaluation of bind affinity between the key ingredients and their drug targets by molecular docking analysis

**Figure 10.**
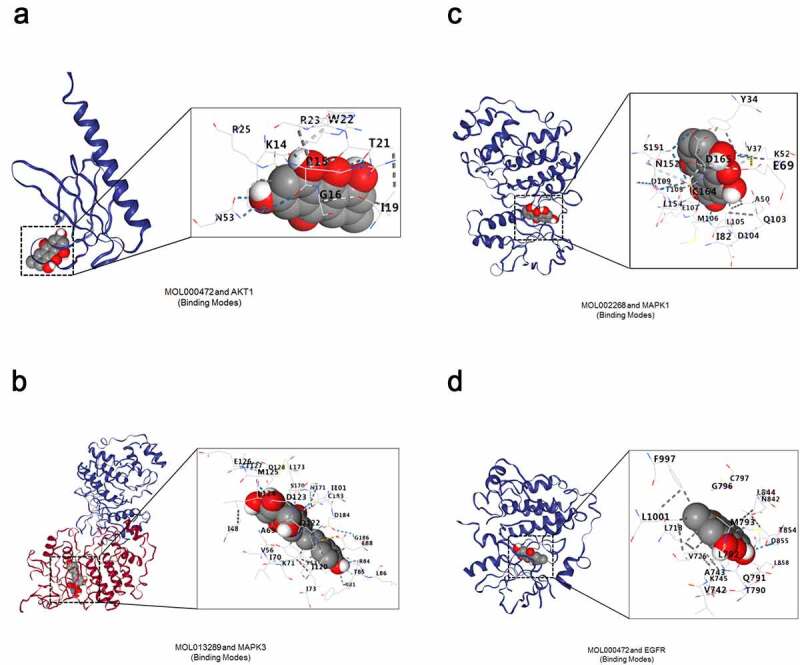
*Molecular docking analysis between seven key ingredients in* polygonum cuspidatum *and the key targets in Akt/ERK/EGFR signaling pathways*. (a) Compound MOL000472 docking with AKT1; (b) Compound MOL002268 docking with MAPK1; (c) Compound MOL013289 docking with MAPK3; (d) Compound MOL000472 docking with EGFR.

Having confirmed the inhibitory effect of *polygonum cuspidatum* on OS cells via disturbance of Akt/ERK/EGFR signaling pathways, we then performed molecular docking analysis between AKT1, MAPK1, MAPK3, EGFR, the key targets involved in the above signaling pathways, and the seven key ingredients of *polygonum cuspidatum* to further explore its molecular mechanism-of-action against OS. The molecular docking result indicated that seven key ingredients of *polygonum cuspidatum* showed high binding activities to four key targets in general (Tables 1 and 2). Furthermore, the docking result also indicated that Vina scores between ingredient emodin (MOL000472) and AKT1, EGFR, ingredient rhein (MOL002268) and MAPK1 as well as between ingredient polydatin (MOL013289) and MAPK3, were high relative to those between others, implying important roles of these three ingredients in delivering the inhibitory effect of *polygonum cuspidatum* on OS cells (Figure 10(a–d)).

**Table 1.** Vina scores of compound-target molecular docking (kcal/mol)

ID number	AKT1(1UNR)	MAPK1(2OJG)	MAPK3(4QTB)	EGFR(3POZ)
MOL012744	−5.9	−8.1	−8.0	−8.0
MOL000472	−7.4	−9.6	−9.2	−8.8
MOL000008	−6.8	−8.9	−8.5	−8.4
MOL002268	−7.0	−9.9	−9.2	−8.7
MOL000492	−6.5	−9.1	−8.7	−8.3
MOL000098	−6.4	−8.9	−8.7	−8.6
MOL013289	−6.6	−8.7	−9.7	−8.1

**Table 2.** Cavities’ sizes of compound-target molecular docking

ID number	AKT1(1UNR)	MAPK1(2OJG)	MAPK3(4QTB)	EGFR(3POZ)
MOL000472	120	1098	916	605
MOL000008	120	1098	885	605
MOL002268	138	1098	916	605
MOL000492	120	1098	916	605
MOL000098	120	1098	916	605
MOL013289	186	1098	916	605

## 4. Discussion

In recent years, network pharmacology has been increasingly employed to explore the action mechanism of TCM [27–30]. In this study, we set out to predict the potential drug targets of *polygonum cuspidatum* by utilizing the key seven ingredients we have previously identified by UPLC-MS/MS [23], which uncovered a potential pharmacological activity of *polygonum cuspidatum* against cancer. To further untangle the complex associations among drug targets, we then constructed a PPI network based on *polygonum cuspidatum*- and OS-associated targets. According to topo-parameters of DC, BC and CC of the network, 250 core targets were established to be involved in pharmacological activities of *polygonum cuspidatum* in OS. Cell proliferation and apoptosis were established to be most affected by *polygonum cuspidatum* treatment. Meanwhile, the most affected signaling pathways includes MAPK, PI3K-Akt and ErbB pathways, which suggests that the suppressive effect of *polygonum cuspidatum* on OS cell growth is attributed to disturbance of these signaling pathway.

In line with the above *in silico* analysis results, our subsequent Western blotting results showed that the levels of p-Akt (T308 & S473), p-ERK (T202/Y204) and pan-EGFR, the key factors involved in the above signaling pathways, were all significantly reduced upon *polygonum cuspidatum* treatment. Furthermore, recent studies have shown that polydatin, a key ingredient from *polygonum cuspidatum*, could suppress the proliferation and metastasis of non-small cell lung cancer (NSCLC) cells. It could also induce apoptosis in laryngeal and cervical cancer cells [31]. Quercetin, another key ingredient contained in *polygonum cuspidatum*, exerts its cytotoxic effects on prostate cancer by impairing cell survival and anti-apoptotic pathways [32].

To investigate the molecular mechanism-of-action of *polygonum cuspidatum* on OS, we conducted molecular docking analyses among key drug targets and key ingredients involved in the most disturbed signaling pathways. The docking results showed good binding affinity between emodin and AKT1/EGFR, rhein and MAPK1, as well as polydatin and MAPK3, suggesting critical roles of these three ingredients in delivering the pharmacological activities of *polygonum cuspidatum* via Akt/ERK/EGFR signaling pathways. In agree with our molecular docking results, emodin has been shown to induce apoptosis of hepatocellular carcinoma cell via PI3K/Akt and MAPK pathways *in vitro* and *in vivo* [33]. Also, emodin could trigger autophagy and apoptotic cell death in NSCLC cells via Akt/mTOR and MAPK signaling [34]. Moreover, polydatin was found to exhibit desirable pharmacological activities in proliferation and metastasis inhibition in hepatocellular carcinoma cells by targeting PI3K/Akt pathway [35]. Together, our results support a view that *polygonum cuspidatum* holds a promising potential in OS treatment with its ‘multi-ingredient, multi-target and multi-function’ pharmacological characteristics (Supplementary Figure S1).

## 5. Conclusions

Our results indicated that *polygonum cuspidatum* could impair the key biological processes involving the growth and viability of OS cells, such as apoptosis and cell cycle. Disturbance of these biological processes is likely attributed to the simultaneous inhibition of multiple signaling pathways, including Akt, ERK and EGFR pathways. Furthermore, the molecular docking results showed that the ingredients emodin, rhein and polydatin may play important roles in delivering the pharmacological activities of *polygonum cuspidatum*, while AKT1, EGFR, MAPK1 and MAPK3 could be the potential therapeutic targets for OS treatment.

## Supplementary Material

Supplemental Material

## Data Availability

The datasets used and/or analyzed during the current study are available from the corresponding author on reasonable request.
