# Supplementary figures and images for: Polygonum cuspidatum inhibits the growth of osteosarcoma cells via impeding Akt/ERK/EGFR signaling pathways
Source: Bioengineered. 2022 Feb 7;13(2):2992–3006. doi: 10.1080/21655979.2021.2017679 (PMC8974113; doi:10.1080/21655979.2021.2017679)

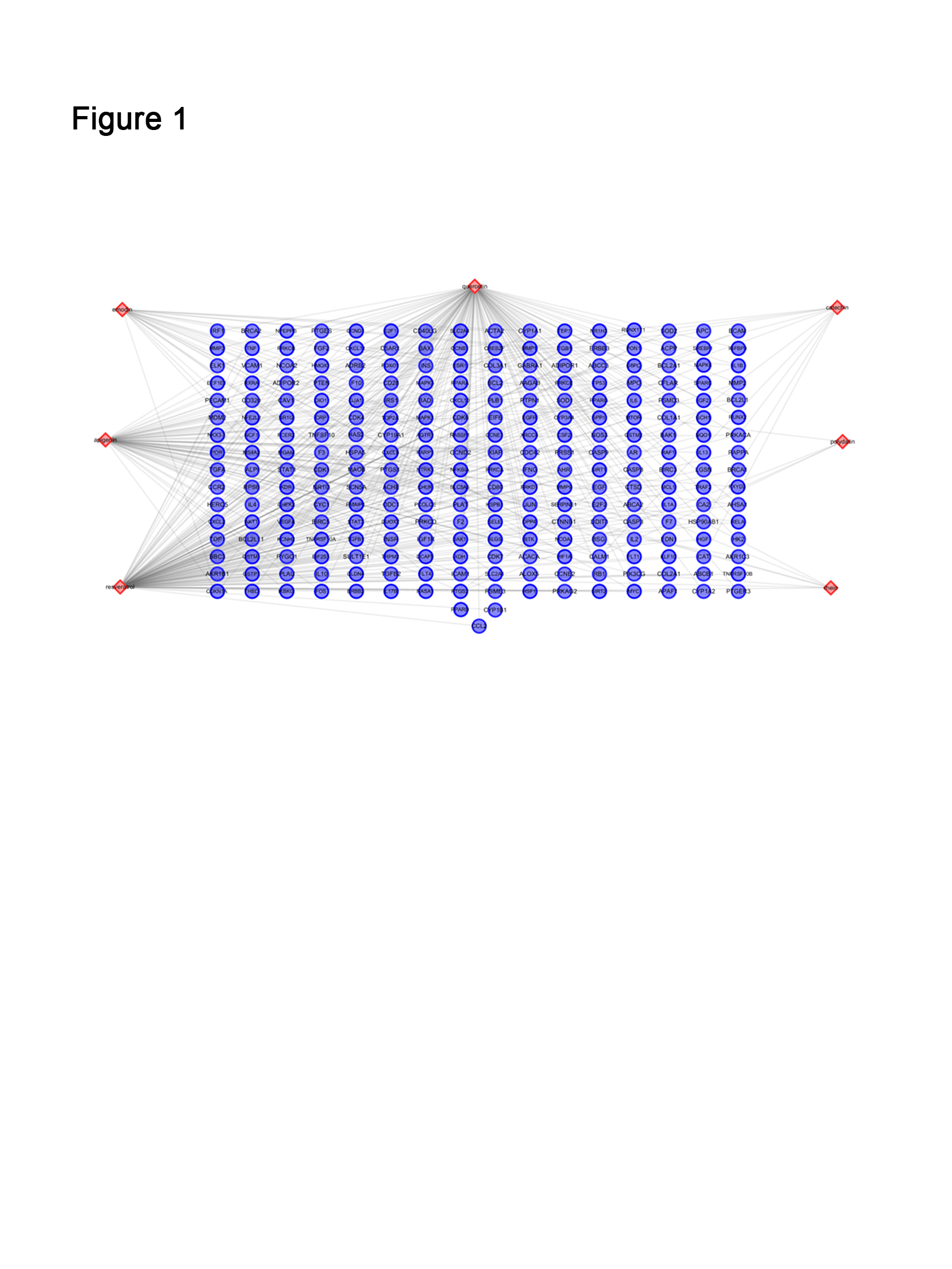

Supplement: Supplemental Material [file KBIE_A_2017679_SM1292.zip › supplementary/Supplementary Figure 1.tif]

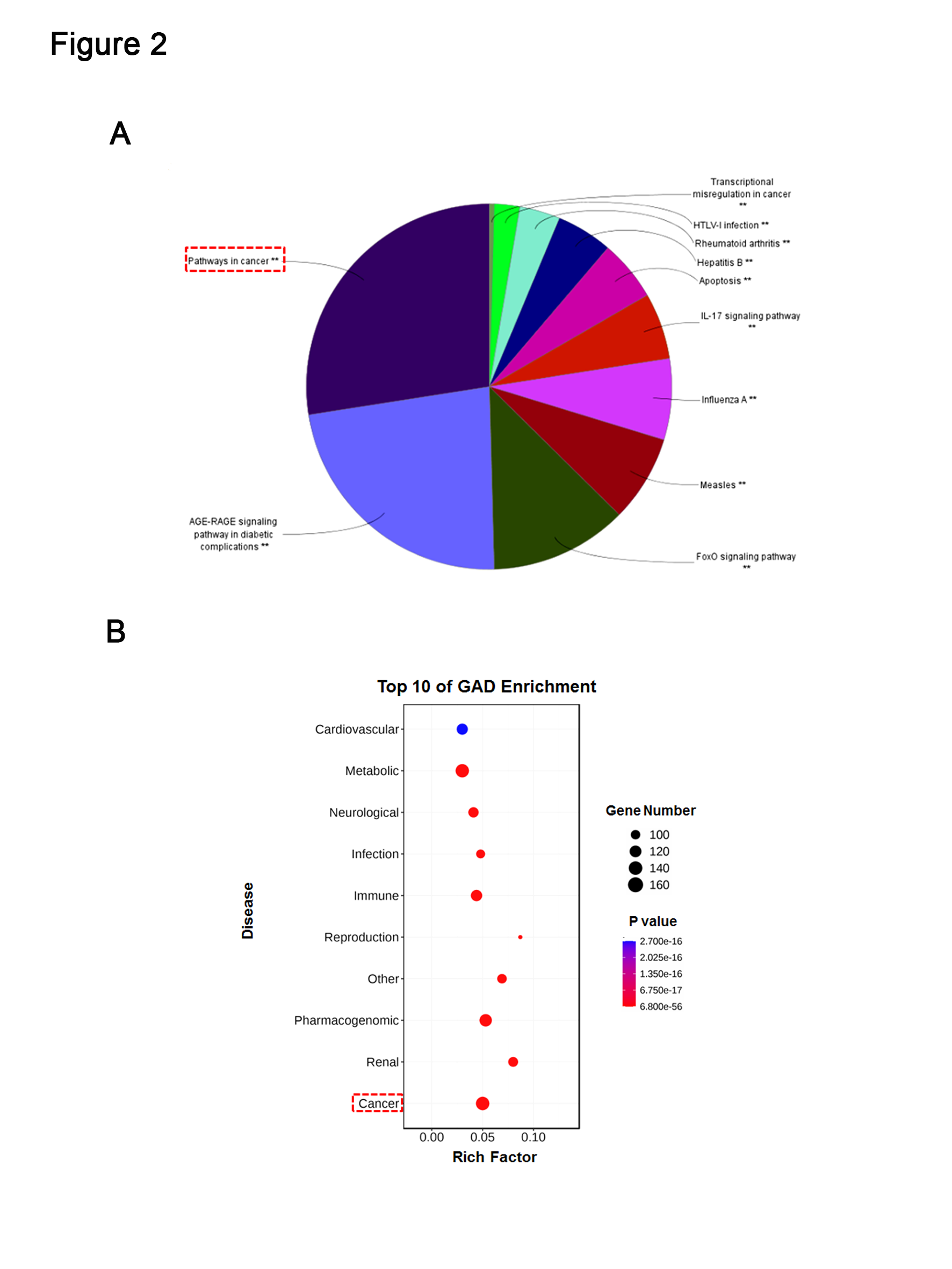

Supplement: Supplemental Material [file KBIE_A_2017679_SM1292.zip › supplementary/Supplementary Figure 2.tif]

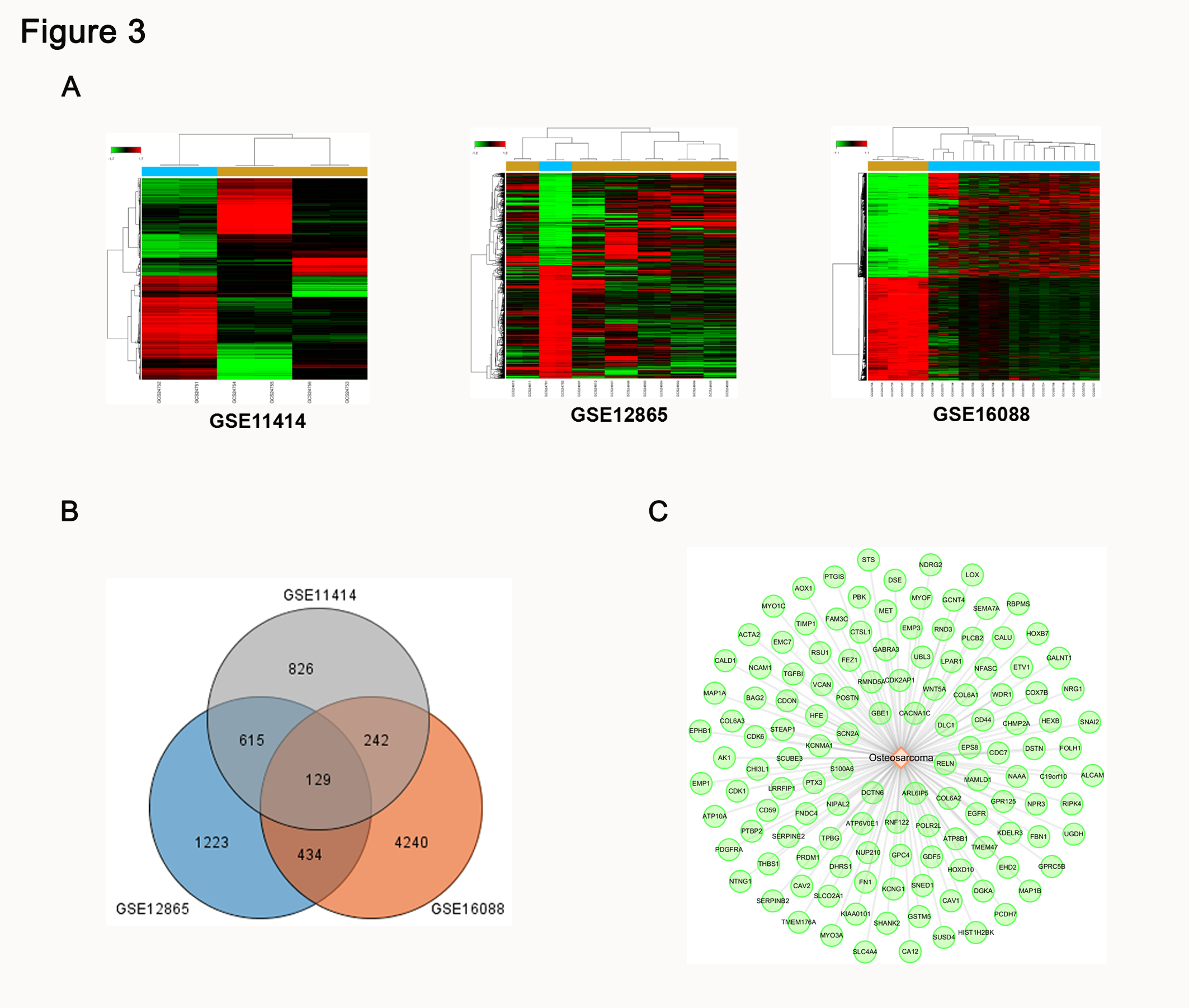

Supplement: Supplemental Material [file KBIE_A_2017679_SM1292.zip › supplementary/Supplementary Figure 3.tif]
